# LO-MLPRNN: A Classification Algorithm for Multispectral Remote Sensing Images by Fusing Selective Convolution

**DOI:** 10.3390/s25082472

**Published:** 2025-04-14

**Authors:** Xiangsuo Fan, Yan Zhang, Yong Peng, Qi Li, Xianqiang Wei, Jiabin Wang, Fadong Zou

**Affiliations:** 1 School of Automation, Guangxi University of Science and Technology, Liuzhou 545006, China; 100002085@gxust.edu.cn (X.F.); 20230202045@stdmail.gxust.edu.cn (Y.Z.); pengyong@gxust.edu.cn (Y.P.); 2 Guangxi Collaborative Innovation Centre for Earthmoving Machinery, Guangxi University of Science and Technology, Liuzhou 545006, China; 3 Engineering Research Center of Advanced Engineering Equipment, University of Guangxi, Liuzhou 545006, China; 4 School of Civil Engineering and Architecture, Guangxi University of Science and Technology, Liuzhou 545006, China; 5 Liuzhou Survey and Mapping Research Institute Co., Ltd., Liuzhou 545005, China; vorpanzer@163.com (J.W.); zoufadond822@163.com (F.Z.)

**Keywords:** multispectral, remote sensing, multilayer perceptron, recurrent neural networks, Omni-Dimensional Dynamic Convolution, large selective convolutional networks

## Abstract

To address the limitation of traditional deep learning algorithms in fully utilizing contextual information in multispectral remote sensing (RS) images, this paper proposes an improved vegetation cover classification algorithm called LO-MLPRNN, which integrates Large Selective Kernel Network (LSK) and Omni-Dimensional Dynamic Convolution (ODC) with a Multi-Layer Perceptron Recurrent Neural Network (MLPRNN). The algorithm employs parallel-connected ODC and LSK modules to adaptively adjust convolution kernel parameters across multiple dimensions and dynamically optimize spatial receptive fields, enabling multi-perspective feature fusion for efficient processing of multispectral band information. The extracted features are mapped to a high-dimensional space through a Gate Recurrent Unit (GRU) and fully connected layers, with nonlinear characteristics enhanced by activation functions, ultimately achieving pixel-level land cover classification. Experiments conducted on GF-2 (0.75 m) and Sentinel-2 (10 m) multispectral RS images from Liucheng County, Liuzhou City, Guangxi Province, demonstrate that LO-MLPRNN achieves overall accuracies of 99.11% and 99.43%, outperforming Vision Transformer (ViT) by 2.61% and 3.98%, respectively. Notably, the classification accuracy for sugarcane reaches 99.70% and 99.67%, showcasing its superior performance.

## 1. Introduction

Contemporary remote sensing technology provides a vast array of information regarding Earth’s surface observations, including high-resolution satellite images and synthetic aperture radar (SAR) data [1,2]. These datasets not only facilitate global-scale ecological monitoring but also support various fields such as forests resource surveys, urban planning, and climate change research [3,4,5]. Accurate mapping of vegetation cover is particularly crucial in tasks such as ecological protection, agricultural monitoring, disaster early warning, and infrastructure development [6,7,8,9]. Consequently, obtaining high-quality, real-time remote sensing data is essential for ensuring the accuracy of vegetation cover mapping. The continuous innovation in remote sensing technology has significantly enhanced the precision of vegetation cover mapping [10]. Alongside the ongoing optimization of remote sensing sensors, data processing algorithms, and deep learning techniques [11,12,13], the capabilities for acquiring and processing remote sensing data have been markedly improved, leading to substantial advancements in the accuracy of vegetation cover mapping.

Several key machine learning algorithms are widely used in the classification processing of remote sensing images, including support vector machine (SVM) [14], random forest (RF) [15], and K-nearest neighbor (KNN) [16]. Support vector machines (SVMs) excel in finding optimal hyperplanes for data classification, particularly in linearly separable scenarios, but struggle with noisy and imbalanced datasets in remote sensing. Random forests (RFs) mitigate overfitting by aggregating multiple decision trees, ensuring stable predictions, yet they may underperform with high-dimensional data due to irrelevant feature selection. K-nearest neighbor (KNN) is simple and intuitive but suffers from high computational complexity with large datasets, making it inefficient for extensive remote sensing data. These limitations hinder their direct and efficient application in such contexts.

In remote sensing image classification, deep learning has achieved remarkable progress, offering significant advantages over traditional methods. It excels at extracting complex, high-level features from large datasets, demonstrates superior feature selection accuracy, and effectively handles noise with strong adaptive capabilities. It is worth noting that in recent years, the field of deep learning has been developing rapidly and changing day by day, and its application in the classification of vegetation cover has become more widespread, becoming the mainstream research and application method in this field [17,18,19]. Convolutional neural network (CNN), a popular neural network, has seen widespread usage in remote sensing categorization. The research of Teja Kattenborn et al. showed that CNN’s ability to exploit spatial patterns particularly enhances the value of very high spatial resolution data, especially in the vegetation remote sensing domain [20]. Xiankun Sun et al. coupled CNN with SVM for an empirical investigation of volcanic ash cloud categorization for the Moderate Resolution Imaging Spectrometer (RSI) [21]. Linshu Hu et al. introduced a CNN-based method for volcanic ash cloud classification, utilizing an end-to-end IE neural network to tackle the scarcity of labeled data in low-light remote sensing. Results showed that RSCNN surpasses traditional IE techniques, achieving a higher structural similarity index and peak signal-to-noise ratio [22]. But CNN mainly relies on local receptive fields, making it difficult to capture global contextual information in images, while land cover classification in remote sensing images often relies on global spatial relationships [23]. Since the birth of the Transformer [24], it has gradually emerged among many models due to its powerful sequence modeling capability and simultaneous perception of the global information of the input sequence. It utilizes the attention mechanism to establish global dependencies between inputs and outputs, while positional encoding effectively addresses the challenge of representing sequence elements’ relative or absolute positional relationships. In 2020, Google proposed the Vision Transformer (ViT) [25], which is a milestone for the application of the Transformer in computer vision, successfully integrating the Transformer architecture into the image classification model. Hong et al. introduced a new backbone network, SpectralFormal, that mines spectral local sequence information from nearby bands of hyperspectral pictures to build component spectral embeddings [26]. Meiqiao Bi et al. proposed ViT-CL, a Vision Transformer (ViT)-based model integrated with supervised contrastive learning, extending the self-supervised contrastive approach to a fully supervised framework. This method leverages label information in the embedding space of remote sensing images (RSIs), enhancing robustness against common image corruptions [27]. However, multispectral remote sensing data contain multiple spectral bands, and ViT may not be able to fully utilize this additional spectral information.

Multilayer Perceptron (MLP) and Recurrent Neural Networks (RNNs) have achieved good results in remote sensing image classification. MLP is the classical nonparametric machine learning method and the most widely used neural network for time series data prediction, aiming to learn the pixel-level nonlinear spectral feature space. IR Widiasari et al. predicted flood events based on rainfall time series data and water level using MLP networks [28]. Z Meng suggested a spectral–spatial MLP (SS-MLP) architecture that employs matrix transpositions and MLPs for spectral and spatial perception in the global receptive field, capturing long-term relationships and extracting more discriminative spectral–spatial characteristics [29]. Ce Zhang et al. proposed an integrated classifier MLP-CNN from the complementary results obtained from CNN based on deep spatial feature representation and MLP based on spectral discrimination [30].

Recurrent neural networks (RNNs) have shown significant potential in analyzing and processing time-series data from multispectral remote sensing images. C. Qi et al. proposed the use of a long-short-term memory network model (LSTM) to invert key water parameters in the Taihu Lake region [31]. As a variant of RNN, LSTM effectively addresses the gradient vanishing problem by incorporating memory units in each hidden layer neuron, enabling controlled storage of time-series information. Each unit utilizes adjustable gates—forget gate, input gate, candidate gate, and output gate—to determine which past and present information to retain or discard, granting the RNN network long-term memory capabilities. G. Wu et al. proposed a new three-dimensional Softmax-guided bi-directional GRU network (TDS-BiGRU) for HSI classification [32]. The processing time can be significantly reduced by utilizing bi-directional GRU for sequence data. Both GRU and LSTM are variants of RNN; GRU, as a variant of LSTM, synthesizes the forget gate and input gate into a single update gate. It likewise mixes cell states and hidden states, plus a few other modifications. The final model is simpler than the standard LSTM model and is also a very popular variant [33].

However, MLP and RNN have certain limitations in multispectral remote sensing image classification [34,35]. MLP processes spectral bands independently, failing to capture their intrinsic connections and synergistic information, which can lead to misclassification or low accuracy in multispectral analysis. For instance, it struggles to integrate correlations between bands reflecting vegetation’s water and chlorophyll content. Similarly, RNN, designed for sequence classification, is limited in extracting spatial information from images. Multispectral remote sensing images contain rich spatial details, such as feature shapes, textures, and neighboring pixel relationships. However, RNN struggles to leverage these spatial differences, making it challenging to accurately classify closely adjacent features with similar spectral characteristics, such as buildings and roads.

In recent years, many scholars have proposed a number of combined model approaches to further improve the accuracy of vegetation cover. DuF et al. introduced an efficient MLP-assisted CNN network with local–global feature fusion for hyperspectral image classification (EMACN) [36]. For the study of the fusion of MLP and RNN, K Wei et al. proposed a multilayer perceptron (MLP)-dominated gate fusion network (MGFNet) [37]. A well-designed MGF module then combines multimodal characteristics through controlled fusion stages and channel attention, employing the MLP as a gate to make use of complementary information and eliminate duplicate data.

This paper proposes LO-MLPRNN to improve multispectral image pixel classification by better leveraging contextual information. In the LO-MLPRNN network framework, the band information of hyperspectral images is processed by the parallel fusion of ODC and LSK modules, and the resulting parallel features are fused. Then, the features extracted by the GRU are further mapped to the high-dimensional feature space through the fully connected layer, and the nonlinear characteristics are strengthened by the sigmoid function. Finally, the classification layer is used to achieve fine multispectral image classification.

The main contributions of this paper are:

(1) A multispectral remote sensing image classification model fusing MLP and RNN is proposed, where the powerful nonlinear mapping of MLP is combined with RNN’s ability to process sequence context and long-term dependence. This combination can more comprehensively extract the data features, taking into account the spectral and spatial sequence features to accurately identify the features in remote sensing image analysis.
(2) From the perspective of multi-feature fusion perception, we propose the MLPRNN network architecture that introduces LSK and ODC. At the same time, we incorporate the multi-head attention mechanism in the LSK module. The two modules fully incorporate the spatial and channel positional associations while allowing the lexical features to have richer expressions for better pixel-level image classification, with a comprehensive classification accuracy of up to 99.43%.
(3) The dynamic change analysis of vegetation utilization in the study area was carried out, focusing on the changes in the distribution area of forests and sugarcane during the period of 2019–2023. Changes in forest cover and crop acreage can visualize the relationship between regional economic development and ecological diversity conservation.

This manuscript is organized into several sections. Section 2, titled “section: Materials and methods”, will delineate the materials utilized in the study area, the image processing techniques employed, and the network framework established. Section 3, section: Experiments and Results, will provide a comprehensive account of the experimental procedures conducted and the subsequent interpretation of the results obtained. Section 4, section: Discuss, will critically analyze both the strengths and limitations of the experiments undertaken. Lastly, Section 5, section: Conclusions, will encapsulate the experiments and findings presented in this paper while offering recommendations for future research endeavors.

## 2. Materials and Methods

### 2.1. Overview of the Study Area

**Figure 1:**
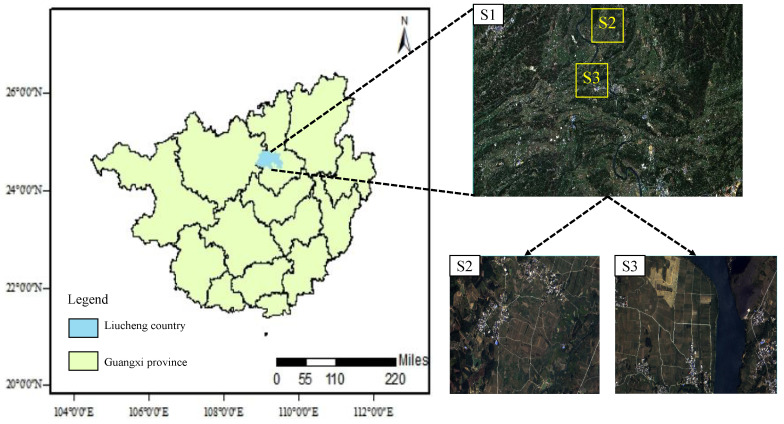
Geographic location of the study area and corresponding remote sensing images, S1 for Sentinel-2 (17 October 2022), S2 and S3 for GF-2 (15 October 2022) image.

Liucheng County, belonging to Liuzhou City, Guangxi Zhuang Autonomous Region, is located in the north-central part of the Guangxi Zhuang Autonomous Region, between longitude 108°36′ E and 108°50′ E and latitude 24°26′ N and 24°25′ N. It is adjacent to Luzhai County in the east, borders Luocheng Mulao Autonomous County and Yizhou District in the west, connects with Liuzhou City Suburb and Liujiang District in the south, and borders Rong’an County and Rongshui County in the north. The maximum horizontal distance between the east and west of the county is 79 km, and the maximum vertical distance between the north and south is 47 km. The total area covers 2114.37 square kilometers. The Liucheng County area belongs to the subtropical monsoon zone, with hot summers and cold winters, four distinct seasons, and abundant light energy and water. The forest cover is wide, and it is very suitable for sugarcane crop cultivation. Therefore, this experiment chooses the Liucheng County area for the study to carry out the following experiments. The specific image is shown in Figure 1.

### 2.2. Remote Sensing Image Pre-Processing

In order to obtain high-quality remote sensing image training samples and accuracy verification samples, the field ground cover sample point data are crucial, and the quality of these data directly determines the accuracy of classification. To obtain the feature types in the study area, the GF-2 satellite data with a resolution of 0.75 m were downloaded from the Jilin-1 website (www.jl1mall.com, accessed on 13 April 2025). The L3C-level image data remote sensing satellite products contain four spectral bands (B, G, R, NIR) and are processed by ortho-correction, geometric correction, and atmospheric correction, with high spatial and spectral resolution. The image map is cropped to obtain S2 (3354 × 2183 pixels) and S3 (3398 × 2092 pixels). The selected areas of S2 and S3 are large sugarcane planting base in Liucheng County, which has a high degree of recognition.

Simultaneously, multispectral images of the Sentinel-2 satellite, which carries a Multispectral Instrument (MSI) containing a total of 12 spectral bands, were downloaded from the ESA website accessed on 8 December 2024 (https://dataspace.copernicus.eu/) in the L2A class. In remote sensing image processing, the selection of relevant bands is a crucial step, as different bands capture different spectral information that can be used for various applications [38]. For example, principal component analysis (PCA) in band selection techniques has been widely studied to optimize the use of multispectral data [39]. The near-infrared (NIR) band is commonly used for vegetation analysis, while the red edge band can provide insights into plant health [40]. The Snap 10.0.0 software was applied to resample the bands at a resolution of 10 m. The ENVI 5.3 software was applied to synthesize the bands at 10 m resolution. Finally, the 12 bands were band-synthesized in sequence in the ENVI software, and the image stitching was completed using the seamless mosaic tool to complete the image cropping and export the image map S1 of Guangxi Liucheng County image data (7926 × 4574 pixels).

This study first uses prior knowledge to label regions of interest (ROIs) in S2 and S3 images to obtain sample library data. The sample library contains 7 categories, with 24,335 and 21,600 samples, respectively, divided into a training set (60%) and testing set (40%). At the same time, based on S2 and S3, S1’s sample library data are obtained, which include 7 categories and 28,570 samples divided into a training set (60%) and testing set (40%). The types and numbers of samples are shown in Table 1.

### 2.3. LO-MLPRNN Network Framework

**Figure 2:**
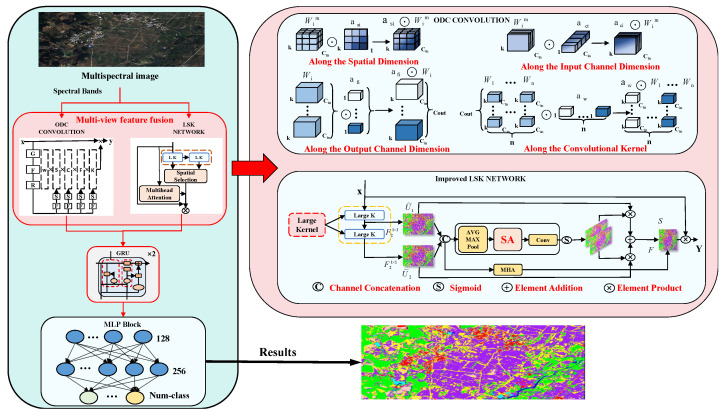
Schematic of LO-MLPRNN architecture for multispectral image classification task.

Aiming at the problem that traditional deep learning algorithms cannot make full use of contextual information in multispectral remote sensing images, this paper proposes the MLPRNN vegetation cover classification algorithm fusing LSK and ODC, called LO-MLPRNN. As shown in Figure 2, the structure mainly consists of an ODC, LSK module, multilayer perceptron (MLP), and GRU. Firstly, the ODC module is used to generate dynamic convolutional features, and the local and global feature representations are further enhanced by the LSK module, which uses multi-scale convolutional operations to capture spatial information under different perceptual fields and integrates the multi-scale features through a specific weighting mechanism to enhance the representational capability. Subsequently, the parallel features generated by the ODC and LSK modules are fused, and the fused features are reorganized into a feature format suitable for RNN input through dimensional transformation. The RNN module performs temporal modeling on the reshaped spectral features to generate hidden layer features that are more expressive of the spectral sequence. The features extracted by the GRU are further mapped to the high-dimensional feature space through a fully connected layer, followed by an activation function to enhance the nonlinear properties, and finally by a classification layer to achieve a fine-grained multispectral image. Finally, the classification layer is used to achieve the refined vegetation classification of multispectral images.

#### 2.3.1. Omni-Dimensional Dynamic Convolution

The ODC introduces a multidimensional attention mechanism with a parallel strategy that learns different attentions of the convolution kernel along all four dimensions of the kernel space [41]. It first uses an attention mechanism to calculate the attention weights for channels, filters, spatial dimensions, and kernels. These weights are used to dynamically adjust the convolutional kernel during the forward pass. The attention mechanism employs a combination of global average pooling, fully connected layers, and activation functions to achieve this. The dynamic convolutional feature of ODConv, which dynamically combines convolution kernels based on the inputs, is able to enhance the extraction of complex features from hyperspectral images. For example, when confronted with different features with different spectral profiles and spatial distributions, ODConv can adjust the convolution operation to better capture these differences, thus improving classification accuracy. Its formula is as follows:

(1) $$y=\left(\alpha_{w1}⊙\alpha_{f1}⊙\alpha_{c1}⊙\alpha_{s1}⊙W_{1}+\ldots +\alpha_{\mathrm{wn}}⊙\alpha_{\mathrm{fn}}⊙\alpha_{\mathrm{cn}}⊙\alpha_{\mathrm{sn}}⊙W_{n}\right)*x$$

**Figure 3:**
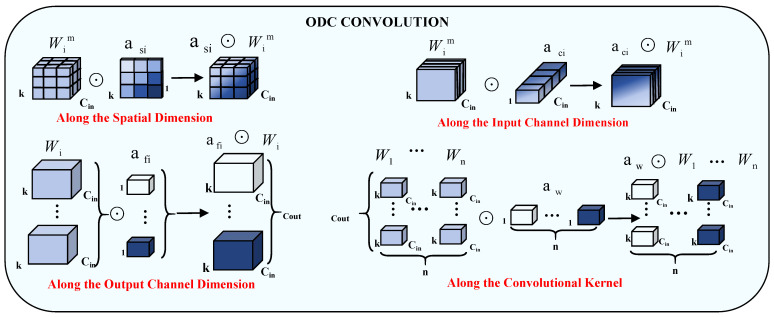
ODConv network architecture schematic.

$\alpha_{\mathrm{wn}}$ denotes a kernel smart multiplication operation along the kernel dimension of the convolution kernel space, assigning an attention scalar to the whole convolution kernel; $\alpha_{\mathrm{fn}}$ denotes a channel smart multiplication operation along the output channel dimension, assigning different attention scalars to the output convolution; $\alpha_{\mathrm{cn}}$ denotes a filter smart multiplication operation along the input channel dimension, assigning a different attention scalar to each of the convolutions; and $\alpha_{\mathrm{sn}}$ denotes a positional smart multiplication operation along the spatial dimension, assigning different attention scalars to convolution parameters at the k × k spatial locations. As shown in Figure 3, redrawn based on article [41], in principle, these four types of attention are complementary to each other and progressively multiply them by the convolution kernel $W_{n}$ in the order of positions, channels, filters, and kernels, such that the convolution operates on all spatial positions of the input image spectral sequences, all input channels, and all kernels. With ODConv’s multi-dimensional attention, the importance of different bands and spatial locations can be automatically learned, highlighting the features that are critical for classification, fully exploiting the need for spectral–spatial features of remotely sensed images to match, and providing performance guarantees for capturing rich contextual cues of remotely sensed images.

#### 2.3.2. Improved Large Selective Kernel Network

**Figure 4:**
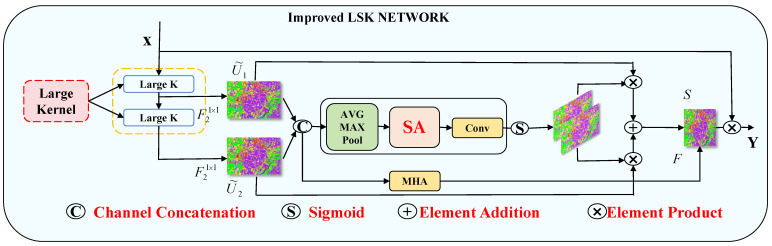
Schematic diagram of the improved LSK network structure.

In order to better handle the contextual information required for vegetation cover classification in remote sensing images, LSK was chosen to be used to connect with ODC in parallel. The large kernel convolution and spatial kernel selection mechanisms of LSK have already taken spatial contextual information into account to a certain extent [42]. The LSK module is a feature enhancement module that combines convolution operations with multi-head attention to further improve the efficiency of spatial feature extraction. It uses two convolutional layers to extract features from the input. This module uses convolutional layers to fuse attention weighted features with the original features. By incorporating a multi-head attention mechanism, the attention weights for feature extraction can be calculated, further enhancing the modeling of contextual information. This allows the model to focus on the most relevant spatial regions. This mechanism mainly reduces and restores the dimensionality of input features by flattening them and passing them through fully connected layers. Its network structure is shown in Figure 4.

When computing attention, each head can look for contextual information related to the current pixel in a wider spatial range (by both macronucleus convolution and spatial kernel selection), depending on the dimension of the feature it is focusing on. Firstly, the feature $\tilde{U_{i}}$ obtained from the same kernel is concatenated with different ranges of receptive fields with the following formula:

(2) $$\tilde{U}=\left[{\tilde{U}}_{1};\ldots ;{\tilde{U}}_{i}\right]$$

Then, effectively extract spatial relationships through channel-based average pooling and max pooling:

(3) $${\mathrm{SA}}_{\mathrm{avg}}=P_{\mathrm{avg}}\left(\tilde{U}\right),{\mathrm{SA}}_{\max}=P_{\max}\left(\tilde{U}\right)$$

where ${\mathrm{SA}}_{\mathrm{avg}}$ and ${\mathrm{SA}}_{\max}$ are the average and maximum pooled spatial feature descriptors. $P_{\mathrm{avg}}$ and $P_{\max}$ denote that, in order to allow information interaction between different spatial descriptors, we pooled the spatially pooled features and used convolution to transform the pooled features into N spatial attentions:

(4) $$\hat{\mathrm{SA}}=F^{2\to \;N}\left(\left[{\mathrm{SA}}_{\mathrm{avg}\;};{\mathrm{SA}}_{\max}\right]\right)$$

Then, for each spatial attention, the individual spatial selection mask is obtained by the sigmoid activation function:

(5) $${\tilde{\mathrm{SA}}}_{i}=\sigma \left({\hat{SA}}_{i}\right)$$

To obtain the attention feature S, the features in the decomposed macroscopic kernel sequence are fused by a convolutional layer after being weighted by their respective spatially selective masks:

(6) $$S=F\left(\sum_{i=1}^{N}\left({\tilde{\mathrm{SA}}}_{i}\;\cdot \;{\tilde{U}}_{i}\right)\right)$$

The proposed structure enhances contextual modeling by integrating a multi-head attention mechanism with feature and channel dimensions of diverse depth convolutional kernels. This allows the model to simultaneously focus on multiple representation subspaces, analyzing relationships between spectral bands, spatial locations, and their interactions. By addressing both spectral and spatial characteristics, the model effectively captures subtle distinctions among bands and their spatial interplay. The key advantage lies in its ability to comprehensively and intricately interpret the intrinsic information within remote sensing images.

#### 2.3.3. Multilayer Perceptron Recurrent Neural Networks

**Figure 5:**
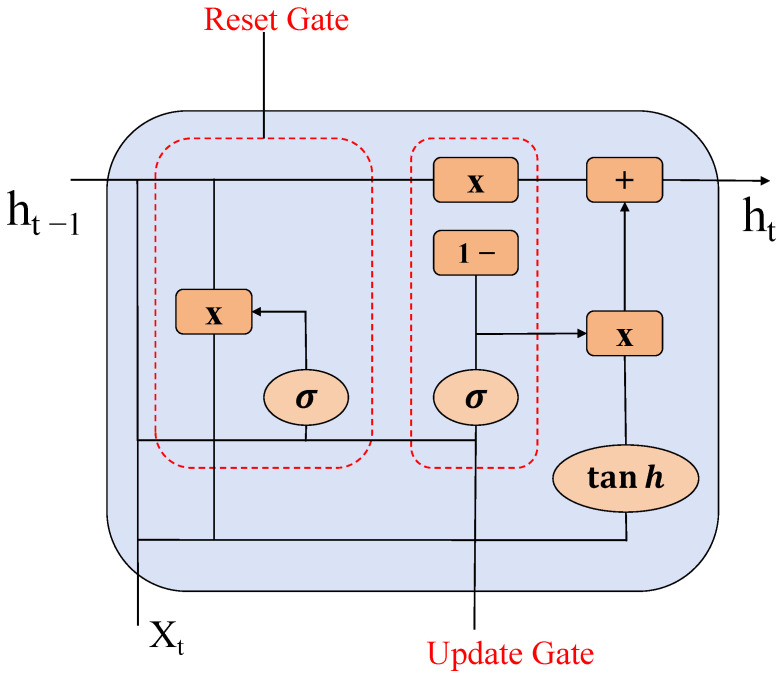
GRU structure.

In this study, we introduce a network architecture that integrates GRU with MLP. GRU is a variant of RNN designed to address the issues of vanishing and exploding gradients that are commonly encountered in traditional RNNs during the training phase. The GRU architecture incorporates components such as update gates and reset gates. In comparison to Long Short-Term Memory (LSTM), another RNN variant, GRU is characterized by a reduced number of parameters, which facilitates expedited training and requires fewer data for effective generalization. At each time step within the input sequence, the update gate regulates the retention of information from the previous hidden state and the integration of new information from the current input. Meanwhile, the reset gate governs the manner in which previous hidden states are combined with the current input to generate new candidate hidden states. Through the continuous execution of these information updating and transferring operations at each time step, the ultimate output is a hidden state representation that encapsulates the entire sequence after processing. The RNN architecture employed in this paper consists of two GRU recurrent layers, with the GRU structure illustrated in Figure 5.

**Figure 6:**
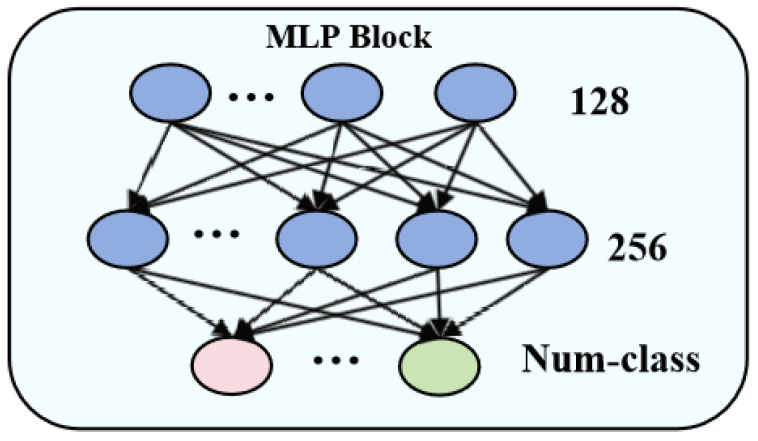
MLP structure.

MLP is a neural network design that consists of an input layer, numerous hidden layers, and an output layer, allowing it to successfully process nonlinear data and adapt to complex data distributions. This model exhibits considerable flexibility and demonstrates strong performance across various tasks, as it does not impose limitations on the relationships among features. The MLP’s capacity to manage nonlinear data without constraining feature relationships, along with its adaptability to complex data distributions, underscores its versatility and efficacy in diverse applications. The specific architecture of the MLP utilized in this study is illustrated in Figure 6. The input layer features a dimensionality of 128, which is expanded to 256 in the hidden layer, while the output layer corresponds to the number of classifications. Notably, the MLP employed in this research implements the ReLU activation function, thereby mitigating the gradient vanishing problem that can arise with traditional sigmoid activation functions during backpropagation.

In general, to handle multispectral images with varying spatial resolutions and spectral band counts, the model primarily employs dynamic convolution layers to adaptively select convolution kernels based on input features. This enables the model to effectively capture spatial information from images of different resolutions. Subsequently, the enhanced LSK module integrates convolution operations with multi-head attention, thereby enhancing spatial feature extraction. This robustness allows the model to adapt to variations in spatial resolution and band count, effectively accommodating image data of diverse resolutions and bands.

In addition, this paper integrates RNN and MLP to process dynamic information in sequential data. The MLP handles fixed-dimensional features extracted by convolutional and pooling layers, enabling global, static feature integration. The RNN captures temporal dependencies in sequences, such as spectral or spatial correlations in multispectral images, by modeling pixel or spectral band sequences. By combining RNN outputs with MLP inputs, the classifier leverages richer features for decision-making. This fusion allows the model to fit more complex data distributions, enhancing the accuracy of multispectral image classification.

### 2.4. Evaluation Indicators

This study assesses multispectral pixel classification through the utilization of three evaluation metrics derived from confusion matrices: Overall Accuracy, Average Accuracy, and Kappa.

Overall accuracy is defined as the proportion of accurate predictions relative to the total number of predictions, expressed as a percentage. The corresponding formula is as follows:

(7) $$\mathrm{Accuracy}=\frac{TP+TN}{TP+FN+FP+TN}$$

In the context of the formula, TP (True Positive) represents the samples accurately identified as belonging to the positive category. Conversely, FP (False Positive) represents the samples that, despite being classified as positive, are actually part of the negative category. TN (True Negative) represents the samples that have been correctly classified as negative, while FN (False Negative) represents the samples that are genuinely in the positive category but have been erroneously classified as negative.

Average accuracy refers to the mean accuracy of a model, which is distinct from overall accuracy. Unlike OA, average accuracy is computed individually for each category and subsequently averaged to yield the final score of the model. The formula for this calculation is as follows:

(8) $$AA=\frac{1}{n}\sum_{i=1}^{n}\frac{TP_{i}+TN_{i}}{TP_{i}+TN_{i}+FP_{i}+FN_{i}}$$

The Kappa coefficient, also known as Cohen’s kappa, is a metric used to evaluate the accuracy of a classifier or assessment system by measuring the agreement between predicted and actual results in a test. It categorizes errors into two types: random errors, akin to arbitrary guessing, and classification errors, where a sample is incorrectly assigned to a different category. The Kappa coefficient ranges from [−1, 1], with values below 0 indicating worse agreement than random chance. Its formula is as follows:

(9) $$\mathrm{Kappa}=\frac{P_{l}-P_{x}}{1-P_{x}}$$

In the formula, $P_{l}$ denotes the precise correspondence between the observed data and the classifier’s predictions, specifically the ratio of instances in which the classifier’s predicted outcome aligns perfectly with the actual outcome. Conversely, $P_{x}$ signifies the probability that the classifier’s predicted classification will coincide with the actual situation, resulting in an identical outcome.

### 2.5. Loss Function

In this study, cross-entropy has been selected as the loss function due to its efficacy in remote sensing image classification tasks, as it enhances the model’s ability to differentiate among various categories. The minimization of the loss function can be achieved through optimization techniques such as gradient descent, which contributes to the model’s accuracy. The cross-entropy loss function evaluates the predicted class of each pixel against the target class using the following expression:

(10) $$L=\frac{1}{N}\sum_{i}L_{i}=-\frac{1}{N}\sum_{i}\sum_{c=1}^{M}y_{ic}\log\left(p_{ic}\right)$$

In the given formula, *M* represents the total number of categories. The variable $y_{ic}$ signifies the sign function, which assumes a value of 1 if the actual category of sample *i* corresponds to category *c* and 0 in all other cases. Additionally, $p_{ic}$ denotes the predicted probability that sample *i* is classified as belonging to category *c*.

### 2.6. Experimental Environment

The experiments presented in this study utilize ENVI software to acquire coordinate point data from the region of interest, which are subsequently exported as a text file for use as training data. During the model training process, a batch size of 32 is employed, alongside a maximum iteration limit of 100 and a learning rate decay factor of 0.9. The experimental code is developed in Python 3.7 and executed using PyTorch version 1.12.0. The training environment comprises a Windows 10 operating system, a 12th Generation Intel(R) Core(TM) i5-8500 processor (Intel, Santa Clara, CA, USA), and an NVIDIA GeForce RTX 3060 GPU (Intel, Santa Clara, CA, USA).

## 3. Results

**Figure 7:**
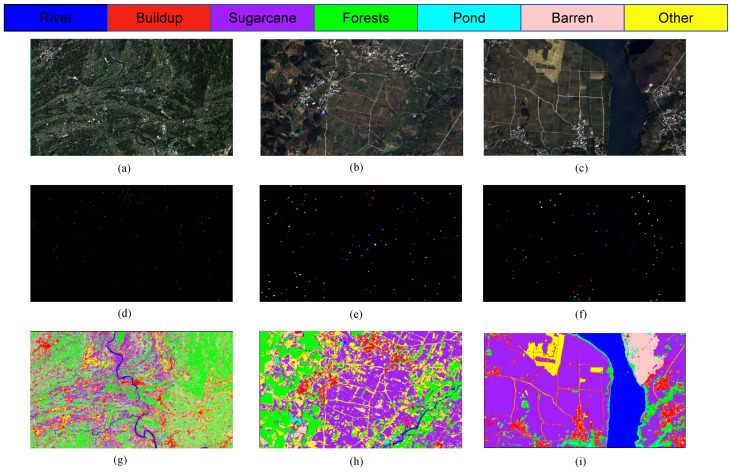
Comparison of ground truth values: (**a**) S1 study area original image; (**b**) S2 study area original image; (**c**) S3 study area original image; (**d**) S1 ground truth value; (**e**) S2 ground truth value; (**f**) S3 ground truth value; (**g**) S2 study area classification image; (**h**) S2 study area classification image; (**i**) S3 study area classification image.

In order to demonstrate the performance of the model more intuitively, Figure 7 shows the original images of the study area, partial ROI labels of ground truth values, and the classification images of the model proposed in this study. In this experiment, the ROI labels of the study area were obtained through field investigations and expert experience on high-resolution GF-2 and Sentinel-2 remote sensing images. The specific sample values are shown in Table 1.

### 3.1. Ablation Experiment

To assess the efficacy of the network architecture proposed in this study, ablation experiments were performed utilizing the datasets from the S1 and S2 study areas. The results pertaining to the GF-2 dataset are presented in Table 2. The OA achieved by the combination of MLP and RNN alone is 97.40%, indicating satisfactory performance. This outcome can be attributed to the dataset’s four-band imagery, which facilitates enhanced feature learning and maintains a higher level of accuracy. The ODC module does not significantly alter the overall accuracy, yielding an OA of 97.24%. The attention mechanism inherent to the ODC may not effectively capture the intricate relationships among the input features of the MLPRNN across time series, thereby limiting its capacity to enhance feature representation and improve accuracy substantially.

In contrast, the introduction of LSK alone results in an OA of 98.99%, alongside improvements in the Average Accuracy (AA) and Kappa statistics, suggesting that LSK effectively addresses the challenges posed by complex sequences. However, the addition of LSK, which employs multiple attention heads, may inadvertently lead to an overemphasis on specific local features, resulting in a slight decline in accuracy, with an OA of 98.88%. The combination of ODC with an unimproved LSK yields an OA of 97.76%, with all validation metrics showing enhancement compared to the MLPRNN, thereby demonstrating the synergistic benefits of this combination for improving image classification performance.

The fully integrated LO-MLPRNN was subsequently evaluated, achieving an OA of 99.11%, which represents a 1.71% improvement over the MLPRNN. The AA reached 99.01%, reflecting a 2.17% enhancement, while the Kappa statistic increased to 0.9892, an improvement of 0.0205, thereby indicating superior performance.

**Figure 8:**
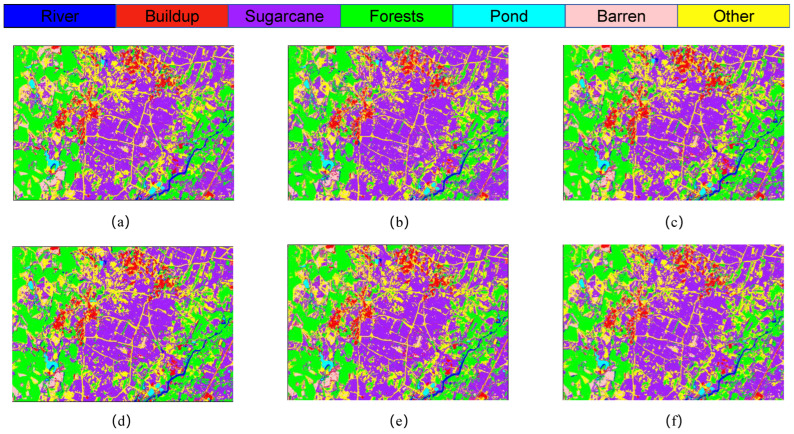
Classification results of LO-MLPRNN using different module combinations for the S1 study area dataset. (**a**) MLPRNN. (**b**) LO-MLPRNN (odc). (**c**) LO-MLPRNN (lsk). (**d**) LO-MLPRNN (improved lsk). (**e**) LO-MLPRNN (odc+lsk). (**f**) LO-MLPRNN (odc+improved lsk).

**Figure 9:**
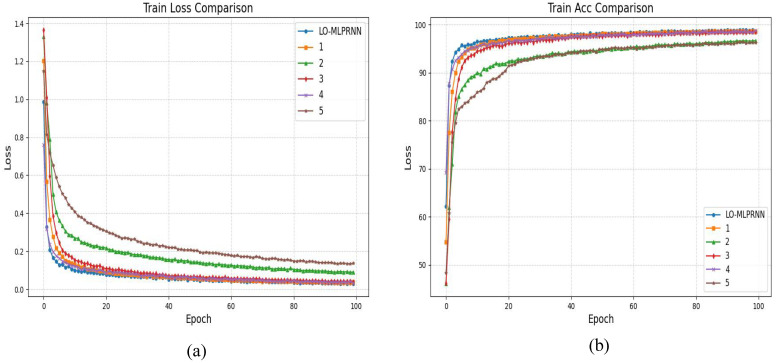
The variation in loss function and accuracy with iteration times in the ablation experiment of S2 research area (**a**) trainloss (**b**) trainacc.

Figure 8 presents the classification outcomes for various combinations of module pairs applied to the GF-2 study area dataset, which includes categories such as forests, sugarcane, and barren land. Comparing Figure 8a, it is observed that the inclusion of the ODC module alongside the LSK module enhances the accuracy of sugarcane classification. However, when the LSK and ODC modules are integrated in parallel, the classification results depicted in plots Figure 8c,d do not achieve the same level of accuracy in identifying built-up areas as plot Figure 8e. Notably, the comprehensive LO-MLPRNN model demonstrates superior discriminative capabilities compared to any other module combination. The primary challenge in classifying this region arises from the overlap between barren land and sugarcane; nevertheless, the complete LO-MLPRNN model exhibits the most effective performance, characterized by significant denoising capabilities. The changes in loss function and accuracy with the number of iterations during the training process are shown in Figure 9.

The experimental findings pertaining to the Sentinel-2 dataset are presented in Table 3. Following the expansion of both the study area and the number of spectral bands, the overall accuracy (OA)of the MLPRNN was recorded at 92.93%. This result suggests that an increase in the number of bands necessitates a more intricate model architecture to effectively manage the data, as there exists a notable correlation among the 12-band dataset that may hinder the simple model’s ability to extract and learn essential features. As additional modules are incorporated, there is a corresponding enhancement in accuracy. Notably, the accuracy of the MLPRNN when integrated with multi-head attention exhibits a substantial improvement, achieving an OA of 98.62%, an average accuracy (AA) of 98.63%, and a Kappa coefficient of 98.39%. This underscores the advantages of employing multi-head attention in the classification of multi-band remote sensing imagery. Ultimately, after the integration of all modules, the OA reached 99.43%, reflecting a 6.4% increase compared to the baseline MLPRNN, with both AA and Kappa also demonstrating significant improvements.

**Figure 10:**
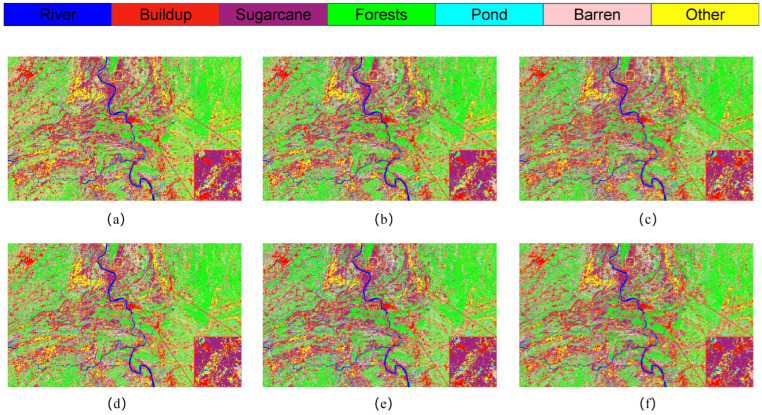
Classification results of LO-MLPRNN using different module combinations for the S1 study area dataset. (**a**) MLPRNN. (**b**) LO-MLPRNN (odc). (**c**) LO-MLPRNN (lsk). (**d**) LO-MLPRNN (improved lsk). (**e**) LO-MLPRNN (odc+lsk). (**f**) LO-MLPRNN (odc+improved lsk).

**Figure 11:**
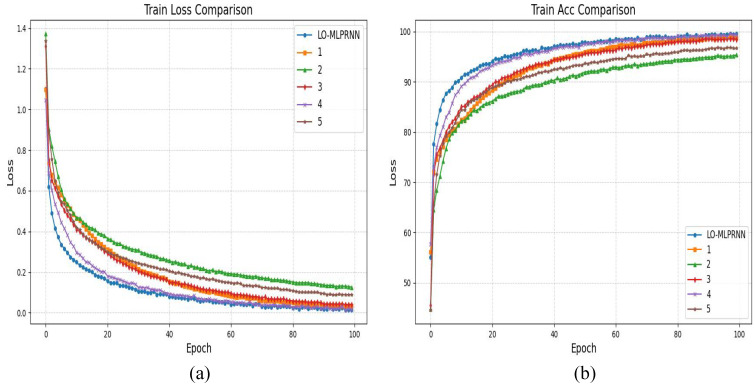
The variation in loss function and accuracy with iteration times in the ablation experiment of S2 research area (**a**) trainloss (**b**) trainacc.

The classification outcomes for various combinations of module pairs within the Sentinel-2 study area dataset are illustrated in Figure 10. The red box in the figure encompasses a portion of the S1 segment of the study area, which includes diverse land types such as sugarcane fields, barren land, and ponds. In the context of multiband analysis, as well as across the broader study area, the primary challenge in classification arises from the need to differentiate between sugarcane, barren land, and other agricultural crops. This difficulty is evident in the red box, which suggests that the MLPRNN model exhibits limitations in accurately classifying barren land and forested areas. Additionally, it is apparent that the inclusion of additional modules contributes to improved differentiation, particularly in more distinct regions such as water bodies and ponds. Notably, the complete LO-MLPRNN model continues to demonstrate a high level of classification accuracy when applied to 12-band multispectral images, indicating effective differentiation capabilities and the potential for further enhancement of network performance. The changes in loss function and accuracy with the number of iterations during the training process are shown in Figure 11.

### 3.2. Multi-Method Comparative Experiments

To elucidate the performance enhancements associated with the proposed model, this study conducts a comparative analysis of the MLPRNN against several established algorithms, including SVM [14], KNN [16], 1D-CNN [20], RNN, ViT [25], SpectralFormer [26], and ViTMLP [43]. The following outlines the configuration parameters for each model:

1. For the SVM, the radial basis function (RBF) is employed as the kernel function, with a penalty factor set to 10 and the decision function shape designated as ‘one-vs-rest’ (ovr).
2. In the case of KNN, the number of neighbors (n_neighbors) is established at 3.
3. For 1DCNN, it consists of a 1D convolutional layer, a bulk normalization layer, ReLU activation function, a 1D maximal pooling layer, a fully connected layer, and an output layer.
4. The RNN architecture consists of two recurrent layers equipped with gating units.
5. For ViT, the structure consists of 5 encoder blocks, each processing a grouped spectral embedding with 64 dimensions. The interior of each encoder block consists of 4 self-attentive layers, a multilayer perceptron (MLP) with 8 hidden layers, and a dropout layer that randomly discards 10% of the neurons.
6. The SpectralFormer model retains the structure described in point (5), with the grouped spectral embedding configured to 2.
7. The ViTMLP model includes the architecture outlined in point (5) with the addition of MLP integration.
8. The LO-MLPRNN module encompasses the MLP module, the RNN module, and the modified LSK and ODC modules. The fully connected layer of the MLP has an input dimension of 128 and an output dimension of 256. The RNN module is structured as a two-layer Gated Recurrent Unit (GRU). The custom LSK module is of the LSKblock type with 12 input channels, while the ODC module is of the ODConv2d type, featuring 12 input and output channels and a convolutional kernel size of 1.

The quantitative classification results of OA, AA, and kappa for datasets S1, S2, and S3, along with per-class accuracies, are shown in Table 4, Table 5 and Table 6. Overall, 1DCNN performs the worst, with OAs of 62.55% and 83.19% in 4-band S2 and S3 regions, and 82.25% in 12-band S1. Limited bands hinder 1DCNN’s ability to extract sufficient spectral features in 4-band regions, while it fails to utilize spatial information effectively in S1. Traditional methods SVM and KNN perform well in S1 and S2, with OAs of 92.82%, 93.46%, 97.63% for SVM and 97.27%, 97.70%, 97.6% for KNN, but struggle with specific classes, e.g., barren in S1 (SVM: 83.84%, KNN: 92.92%). Deep learning models (RNN, ViT, CAF, ViTMLP) show similar performance, demonstrating strong feature extraction capabilities. The proposed LO-MLPRNN achieves the best OA of 99.11%, outperforming all methods. In S2, LO-MLPRNN achieves the highest accuracy for all classes except pond (99.69% vs. SVM’s 100%).

**Figure 12:**
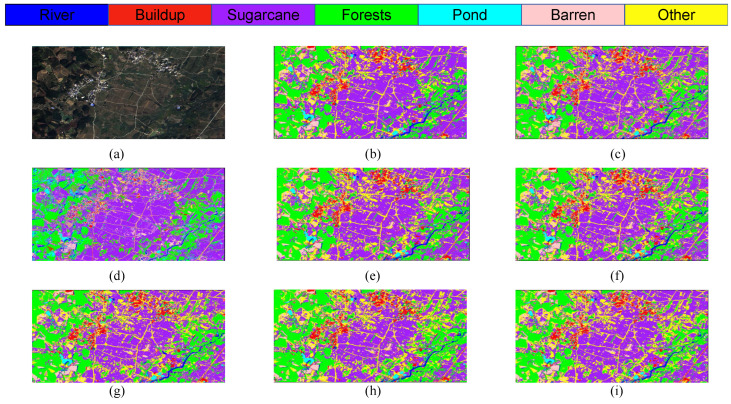
Classification results obtained by different models on S2 data: (**a**) original image. (**b**) SVM. (**c**) KNN. (**d**) 1DCNN. (**e**) RNN. (**f**) ViT. (**g**) CAF. (**h**)ViTMLP. (**i**) LO-MLPRNN.

**Figure 13:**
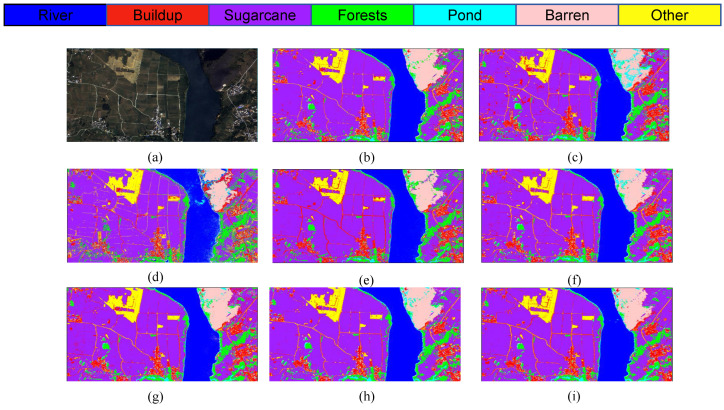
Classification results obtained by different models on S3 data: (**a**) original image. (**b**) SVM. (**c**) KNN. (**d**) 1DCNN. (**e**) RNN. (**f**) ViT. (**g**) CAF. (**h**) ViTMLP. (**i**) LO-MLPRNN.

**Figure 14:**
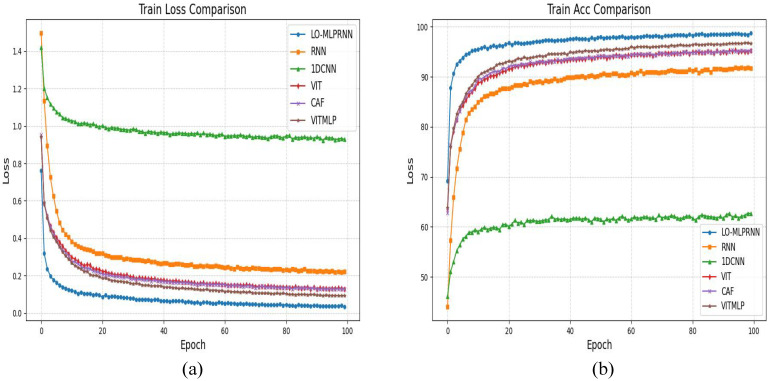
The training loss and accuracy changes of each classification model in the S2 study area: (**a**) trainloss (**b**) trainacc.

The S2 and S3 classification maps obtained through different models shown in Figure 12, Figure 13 and Figure 14 show the training loss and accuracy changes of each classification model in the S2 study area. It is evident that the 1D-CNN model exhibits suboptimal performance in the overall classification within the study area. Conversely, the models ViTMLP, CAF, and LO-MLPRNN demonstrate enhanced classification outcomes. Notably, LO-MLPRNN exhibits superior classification accuracy in specific regions, particularly in its ability to differentiate between the challenging pond and river categories, while also effectively minimizing misclassifications of the barren category. The classification graph distinctly illustrates that LO-MLPRNN outperforms the other algorithms in terms of accuracy and clarity in detailed performance metrics.

**Figure 15:**
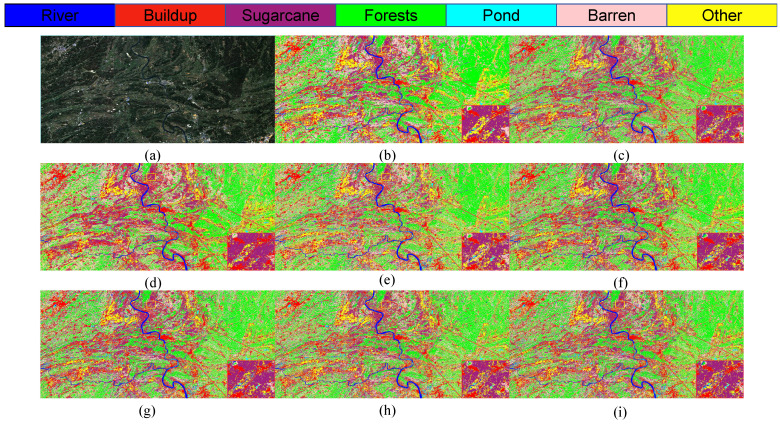
Classification results obtained by different models on Sentinel-2 data: (**a**) original image. (**b**) SVM. (**c**) KNN. (**d**) 1DCNN. (**e**) RNN. (**f**) ViT. (**g**) CAF. (**h**) ViTMLP. (**i**) LO-MLPRNN.

**Figure 16:**
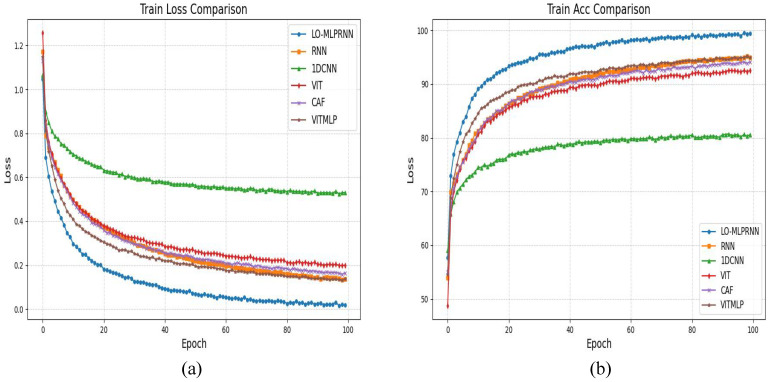
The training loss and accuracy changes of each classification model in the S1 study area: (**a**) trainloss (**b**) trainacc.

As illustrated in Figure 15, the classification outcomes of S1 data across various models indicate that SVM and 1DCNN exhibit notably inferior performance in the selected zoomed-in regions. In contrast, among the other algorithms assessed, the LO-MLPRNN demonstrates superior accuracy in delineating the boundaries of built-up areas and sugarcane categories, with a more pronounced differentiation of blocks. Overall, LO-MLPRNN significantly surpasses the performance of the other algorithms in the context of multispectral image classification tasks. Despite the increased complexity of the proposed model’s architecture compared to other deep learning networks, it achieves substantial enhancements in OA, AA, and Kappa coefficient. Furthermore, the quality of the classification maps produced by LO-MLPRNN is markedly superior to that of the other algorithms, underscoring its considerable significance in this domain. The training loss and accuracy changes of each classification model in the S1 study area are shown in Figure 16.

**Figure 17:**
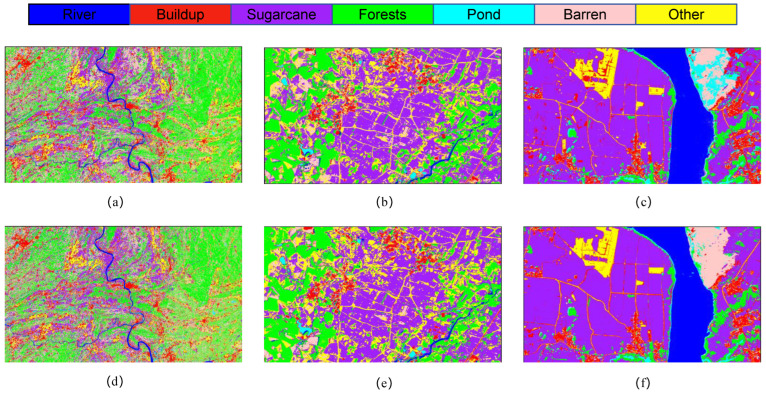
Comparison of classification results between KAN and LO-MLPRNN in three study areas: (**a**) KAN in S1. (**b**) KAN in S2. (**c**) KAN in S3. (**d**) LO-MLPRNN in S1. (**e**) LO-MLPRNN in S2. (**f**) LO-MLPRNN in S3.

To further validate model performance, this study compares LO-MLPRNN with the Kolmogorov–Arnold Network (KAN) [44]. Benefiting from its external similarity to MLP, KAN not only learns features but also optimizes them for higher accuracy. KAN has recently been applied in remote sensing, such as Minjong Cheon’s integration of KAN with pre-trained CNN models for remote sensing (RS) scene classification [45], and H Niu’s proposed multimodal Kolmogorov–Arnold fusion network for interpreting urban informal settlements (UISs) using RS and street-view images [46]. In this experiment, LO-MLPRNN outperforms KAN across all three study areas, as shown in Table 7. KAN achieves accuracies of 90.56%, 91.93%, and 97.77% in S1, S2, and S3, respectively, while LO-MLPRNN demonstrates superior performance, confirming its applicability and high effectiveness in the study areas. The specific classification comparison is shown in Figure 17.

### 3.3. Vegetation Dynamics

In this paper, the Sentinel-2 series images of 12 December 2019, 6 December 2021, and 21 November 2023, downloaded from the ESA website, were used for the study of vegetation dynamics. After preprocessing, the labels were recreated using the 2022 labeled ROIs with prior knowledge and the corresponding txt files were generated. Then, the sample set was randomly divided into training and test sets in the ratio of 6:4 according to the coordinates of the files. Finally, the classification of vegetation dynamics changes in the study area was carried out using the LO-MLPRNN proposed in this paper, focusing on analyzing the changes in the cover of forest and sugarcane.

The classification results of different models for the 2019, 2021, and 2023 sample datasets are shown in Table 8, Table 9 and Table 10. LO-MLPRNN consistently demonstrates superior performance across all five years, achieving OAs of 99.65%, 99.24%, and 99.71%, the highest among all compared models. The improved accuracy on the 2023 dataset may be attributed to its higher spatial resolution or reduced noise, enabling better feature learning. Overall, LO-MLPRNN significantly outperforms other models. Compared to the original RNN, LO-MLPRNN shows improvements of 3.24%, 2.20%, and 0.87% in 2019, 2021, and 2023, respectively. The smaller improvement in 2023 suggests the original RNN already performed well that year. However, the substantial gains in the first two years provide stronger evidence and value for dynamic analysis of forest and other land cover changes over the five-year period. In summary, LO-MLPRNN’s classification performance meets the requirements for analyzing forest utilization changes in the study area of Liucheng County.

**Figure 18:**
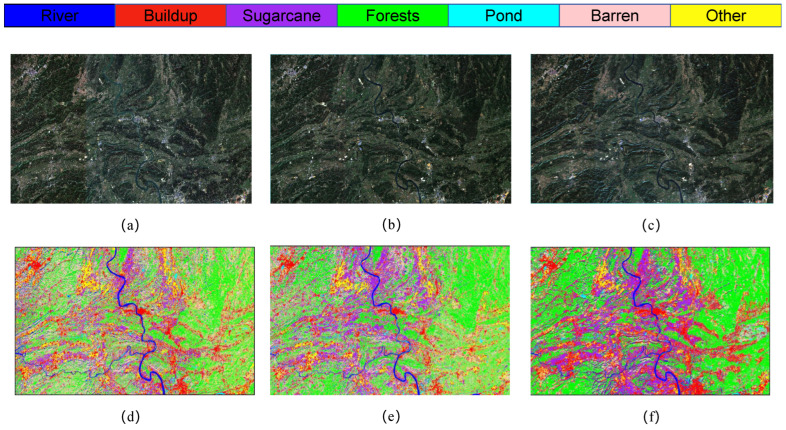
True-color maps of three-phase images: (**a**) 2019, (**b**) 2021, and (**c**) 2023; spatial distribution of features in three-phase images: (**d**) 2019, (**e**) 2021, and (**f**) 2023.

The spatial distribution of vegetation utilization in 2019, 2021, and 2023 is shown in Figure 18. Forest areas have expanded annually, particularly from the periphery toward the center, reflecting the local government’s emphasis on ecological protection and forest management. Sugarcane cultivation is concentrated in the southwest and central regions, benefiting from fertile soil, favorable climate, and ample water resources. Its area has grown significantly over the five years, driven by agricultural policies and market demand. Rivers, primarily located in the central area, provide essential water resources for agriculture and ecological balance. Ponds, surrounded by buildup and barren land, likely serve aquaculture or as natural features, while buildup areas have expanded, indicating urbanization and population growth. Overall, the spatial distribution of forest utilization has become more balanced and rational, demonstrating sustainable and efficient management. This supports forest growth, ecological improvement, and biodiversity conservation in the region.

Table 11 presents the patterns of vegetation utilization and the rate of change in vegetation use within the study area over the past five years. Notably, the predominant type of vegetation utilization has shifted from barren land to forested areas. This transformation may be attributed to a series of ecological restoration and afforestation initiatives undertaken by the local government aimed at enhancing biodiversity and improving the ecological environment.

The significant increase in sugarcane area from 2019 to 2023 may be attributed to the rising demand for sugar and sugar-based products, as well as government support measures aimed at encouraging farmers to expand sugarcane cultivation. The increasing area of riverine vegetation indicates a growing recognition among local communities of the necessity for reforestation and the cultivation of crops, thereby highlighting the importance of water resource management.

**Figure 19:**
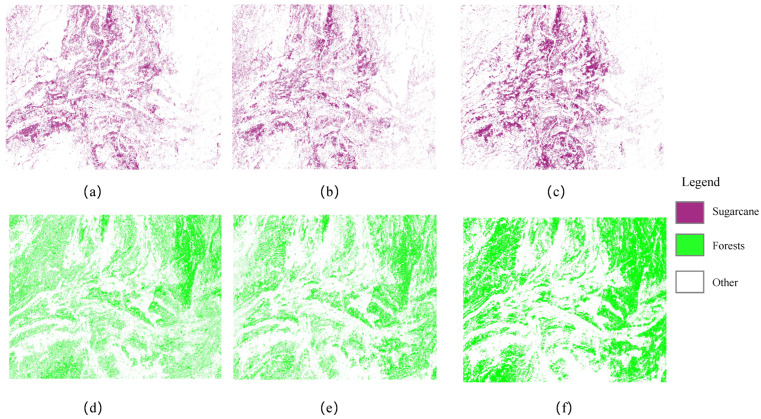
Spatial distribution of sugarcane: (**a**) 2019, (**b**) 2021, and (**c**) 2023 in the three-phase image; spatial distribution of forests: (**d**) 2019, (**e**) 2021, and (**f**) 2023 in the three-phase image.

This study emphasizes the geographical distribution of forests and sugarcane, as represented in Figure 19. The graphic clearly shows that woodlands surround the research area. This distribution can be linked to the abundance of forest resources, which play an important role in preserving ecological balance, protecting water sources, and moderating soil erosion. Sugarcane agriculture is centered predominantly in the study area’s western and central areas. Notably, the area allocated to sugarcane farming and the quantity of forest cover have grown significantly in recent years. The enormous cultivation of sugarcane represents not only the increase in and diversification of agricultural production but also the government’s strategic planning and activities targeted at increasing agricultural output and maintaining food security. Concurrently, the growth in forest area demonstrates the local government’s dedication to ecological protection and forest resource management, as well as the implementation of numerous ecological restoration and afforestation projects. The simultaneous development of these two forms of land use demonstrates a harmonic balance between agricultural productivity and environmental protection in the studied region.

**Figure 20:**
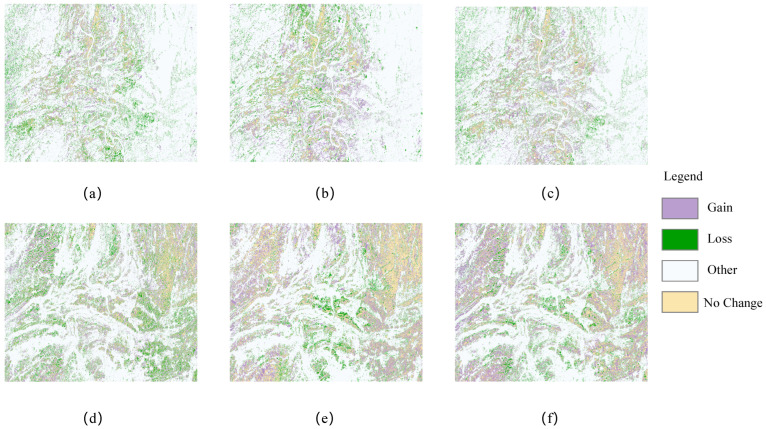
Dynamics of suagrcane in 2019–2021 (**a**), 2021–2023 (**b**), and 2019–2023 (**c**); dynamics of forest in 2019–2023 (**d**), 2021–2023 (**e**), and 2019–2023 (**f**).

The dynamic changes in sugarcane and forest areas from 2019 to 2023 are illustrated in Figure 20. During this period, the forest area decreased from 908.91 km^2^ in 2019 to 841.86 km^2^ in 2022, before significantly increasing to 1225.16 km^2^ in 2023. Specifically, forest area declined by 7.38% from 2019 to 2021 but surged by 45.53% from 2021 to 2023, resulting in an overall growth rate of 34.79% over the entire observation period (2019–2023), indicating a consistent upward trend.

For sugarcane, the cultivated area was 443.96 km^2^ in 2019, slightly increased to 458.48 km^2^ in 2021, and then expanded significantly to 547.25 km^2^ in 2023. This represents a growth of 3.27% from 2019 to 2021 and a substantial increase of 19.36% from 2021 to 2023, with an overall growth rate of 23.27% over the five-year period. The notable expansion of sugarcane cultivation from 2019 to 2023 aligns with expectations, as the region’s fertile soil provides optimal natural conditions for sugarcane production, and the increased cultivation area aims to enhance economic benefits.

## 4. Discussion

This study evaluates the strengths and weaknesses of traditional machine learning algorithms, specifically SVM and KNN, alongside deep learning algorithms including 1DCNN, RNN, Transformers (Vision Transformers—ViT), CAF, and our proposed algorithm, LO-MLPRNN, in the context of multispectral image classification. The findings indicate that the proposed LO-MLPRNN algorithm exhibits a notable enhancement in classification performance. Traditional algorithms such as SVM and KNN demonstrate high efficiency characterized by short training durations while maintaining commendable classification accuracy, even when applied to high-resolution images. However, the classification accuracy of 1DCNN is suboptimal due to the limited information inherent in multispectral images. Conversely, RNNs, while advantageous for processing sequential data and outperforming 1DCNN in classification tasks, struggle to effectively capture long-term dependencies between inputs and outputs. This limitation is addressed by Transformers, which leverage positional encoding to efficiently learn global sequence information.

Our LO-MLPRNN model achieves pixel-level classification accuracy for Sentinel-2 remote sensing images within a multispectral dataset, showcasing superior classification performance. Nonetheless, the model’s requirement for multi-scale feature extraction results in an increased number of parameters and extended computation times compared to single deep learning models, thereby escalating computational costs. Furthermore, the model exhibits reduced efficacy in managing images characterized by complex long-range dependencies, such as those encountered in urban road segmentation scenarios. In instances of detecting irregularly shaped buildings with significant size variations, the feature extraction process may prove inadequate, leading to potential misclassifications or omissions in complex backgrounds and occluded situations.

To enhance the model’s performance and facilitate the handling of larger remote sensing image datasets in future endeavors, we intend to investigate model compression techniques aimed at reducing both the number of parameters and computation times. Additionally, we will consider the integration of spatial attention mechanisms to bolster the model’s capacity to comprehend and classify intricate scenes. Furthermore, we will explore strategies to better amalgamate spatial and spectral information from the images, thereby improving the model’s robustness and accuracy in complex environments and across diverse feature types.

## 5. Conclusions

This study proposes a LO-MLPRNN framework that integrates multi-layer perceptrons and recurrent neural networks to address the issues of insufficient utilization of contextual information and weak adaptability to band feature differences in ground cover classification of multispectral remote sensing images. Through experimental verification in Liucheng County, Guangxi, the classification accuracy based on GF-2 and Sentinel-2 images from 2019 to 2023 reached 99.65%, 99.24%, 99.43%, and 99.71%, respectively, significantly better than the comparative algorithms and demonstrating excellent classification performance. The innovation of this algorithm lies in the parallel integration of ODC and LSK modules, forming a multi-perspective feature fusion mechanism: the LSK module enhances key contextual feature extraction through selective convolution, while the ODC module dynamically adjusts the convolution kernel to adapt to spatially changing features. The model effectively captures temporal features through the GRU layer and then implements feature mapping and classification through the fully connected layer. The experimental results show that this method cannot only stably process multispectral images of different bands but also has strong feature expression ability. Future research will focus on developing lightweight network architectures that balance performance and efficiency in order to expand their applications in a wider range of remote sensing scenarios.

## Figures and Tables

**Table 1:** Sample size of the dataset for the study area Liucheng County.

No.	Class	S1		S2		S3	
		Training	Testing	Training	Testing	Training	Testing
1	River	3033	1011	1059	354	1030	344
2	Buildup	3056	1019	1940	647	1663	555
3	Sugarcane	3078	1027	4426	1476	3343	1115
4	Tree	3040	1014	3921	1308	2394	799
5	Barren	3007	1003	2538	847	4576	1526
6	Pond	2968	990	1648	550	1219	407
7	Other	3243	1081	2715	906	1971	658
Total		21,425	7145	18,247	6088	16,196	5404
		28,570		24,335		21,600	

**Table 2:** Results of ablation experiments of LO-MLPRNN with different module combinations on the GF-2 study area.

No.	Different Methods	Different Module			Metric			Time (s)
		ODC	LSK	Improved LSK	OA (%)	AA (%)	Kappa	
1	MLPRNN	**×**	**×**	**×**	97.40	96.84	0.9687	650.87
2	LO-MLPRNN	✓	**×**	**×**	97.24	97.26	0.9678	1295.37
3	LO-MLPRNN	**×**	✓	**×**	98.99	98.85	0.9878	1264.01
4	LO-MLPRNN	**×**	**×**	✓	98.88	98.77	0.9865	1674.05
5	LO-MLPRNN	✓	✓	**×**	97.76	97.38	0.9730	1737.72
6	LO-MLPRNN	✓	**×**	✓	**99.11**	**99.01**	**0.9892**	1777.36

**Table 3:** Results of ablation experiments of LO-MLPRNN with different module combinations on the Sentinel-2 study area.

No.	Different Methods	Different Module			Metric			Time (s)
		ODC	LSK	Improved LSK	OA (%)	AA (%)	Kappa	
1	MLPRNN	**×**	**×**	**×**	92.93	93.00	0.9175	711.66
2	LO-MLPRNN	✓	**×**	**×**	97.00	97.02	0.9650	1471.94
3	LO-MLPRNN	**×**	✓	**×**	96.97	96.97	0.9647	1707.61
4	LO-MLPRNN	**×**	**×**	✓	98.62	98.63	0.9839	1734.20
5	LO-MLPRNN	✓	✓	**×**	97.89	97.92	0.9754	1979.39
6	LO-MLPRNN	✓	**×**	✓	**99.43**	**99.43**	**0.9934**	2463.88

**Table 4:** Comparison of classification results of different classification methods on S1 2022 dataset.

Class	Different Methods							
	SVM	KNN	1DCNN	RNN	ViT	CAF	ViTMLP	LO-MLPRNN
River	96.43	98.71	92.74	98.94	97.95	98.87	99.10	**100.00**
Buildup	96.85	97.84	91.97	98.16	97.83	98.75	98.72	**99.80**
Sugarcane	93.86	99.12	93.76	99.15	98.60	99.12	98.96	**99.67**
Tree	88.75	95.95	80.96	95.09	93.64	95.35	95.39	**99.30**
Barren	83.84	92.92	62.15	93.47	91.41	93.67	96.10	**97.93**
Pond	**100.00**	99.49	92.95	99.39	98.38	98.65	99.56	99.69
Other	90.19	96.85	62.36	96.42	90.59	90.74	93.76	**99.59**
OA (%)	92.82	97.27	82.25	97.23	95.45	96.40	97.34	**99.43**
AA (%)	92.85	97.27	82.42	97.24	95.49	96.46	97.37	**99.43**
Kappa	0.9162	0.9689	0.7929	0.9677	0.9469	0.9580	0.9689	**0.9934**
Time (s)	84.46	68.62	549.06	612.51	3407.10	3903.70	3354.82	3463.88

**Table 5:** Comparison of classification results of different classification methods on S2 2022 dataset.

Class	Different Methods							
	SVM	KNN	1DCNN	RNN	ViT	CAF	ViTMLP	LO-MLPRNN
River	76.55	95.19	91.02	88.38	98.58	97.63	98.67	**99.52**
Buildup	89.79	93.66	40.87	76.18	92.26	91.23	94.12	**96.44**
Sugarcane	98.91	99.18	90.93	**99.72**	99.57	99.27	98.75	99.70
Tree	**100.00**	99.31	67.91	98.39	97.04	97.24	97.80	99.51
Barren	95.15	97.75	35.73	97.28	99.01	89.83	98.38	**99.92**
Pond	**100.00**	**100.00**	66.14	**100.00**	**100.00**	**100.00**	**100.00**	**100.00**
Other	78.80	95.36	35.80	77.20	88.47	89.80	94.06	**97.97**
OA (%)	93.46	97.70	62.55	92.61	96.50	96.50	97.42	**99.11**
AA (%)	91.32	97.21	61.21	91.03	96.42	96.30	97.40	**99.01**
Kappa	0.9209	0.9723	0.5456	0.9107	0.9579	0.9578	0.9689	**0.9892**
Time (s)	25.92	20.71	464.57	572.97	3036.03	3445.71	3241.52	1777.36

**Table 6:** Comparison of classification results of different classification methods on S3 2022 dataset.

Class	Different Methods							
	SVM	KNN	1DCNN	RNN	ViT	CAF	ViTMLP	LO-MLPRNN
River	**100.00**	95.19	95.24	99.70	**100.00**	**100.00**	99.61	**100.00**
Buildup	90.99	93.66	63.19	89.83	95.24	96.09	93.68	**97.59**
Sugarcane	**100.00**	99.18	92.58	99.73	99.91	99.82	99.46	**100.00**
Tree	**98.24**	99.31	85.25	90.43	99.37	99.24	98.95	97.41
Barren	**100.00**	97.75	98.01	99.97	99.75	95.48	**100.00**	99.86
Pond	84.52	**100.00**	36.50	96.55	98.44	99.91	99.69	**100.00**
Other	**99.84**	95.36	69.81	89.14	95.53	89.80	97.00	98.42
OA (%)	97.63	98.06	83.19	95.88	98.67	98.88	98.28	**99.14**
AA (%)	96.23	97.58	77.23	95.06	98.32	98.64	97.72	**99.04**
Kappa	0.9710	0.9764	0.7938	0.9497	0.9838	0.9863	0.9791	**0.9895**
Time (s)	18.86	15.49	394.67	454.13	2740.03	3029.72	2878.31	1992.13

**Table 7:** Comparison table of classification results between KAN and LO-MLPRNN in three study areas.

Class	S1		S2		S3	
	KAN	LO-MLPRNN	KAN	LO-MLPRNN	KAN	LO-MLPRNN
River	96.50	100	91.78	99.52	100	100
Buildup	93.11	99.80	97.57	96.44	96.21	97.59
Sugarcane	97.72	99.67	97.55	99.70	99.31	100.00
Tree	91.73	99.30	98.69	99.51	97.91	97.41
Barren	84.12	97.93	88.81	99.92	98.12	99.86
Pond	95.68	99.69	98.72	100	94.58	100
Other	75.96	99.59	74.95	97.97	96.29	98.42
OA (%)	90.56	99.43	91.93	99.11	97.77	99.14
AA (%)	90.69	99.43	91.16	99.01	97.49	99.04
Kappa	0.8899	0.9934	0.9027	0.9892	0.9728	0.9895

**Table 8:** Comparison of classification results of different classification methods on the Willow County 2019 dataset.

Class	Different Methods							
	SVM	KNN	1DCNN	RNN	ViT	CAF	ViTMLP	LO-MLPRNN
River	97.92	99.40	89.70	98.84	96.96	98.71	99.07	**100.00**
Buildup	97.05	96.66	89.15	98.59	96.46	97.70	98.88	**99.96**
Sugarcane	98.24	**99.22**	98.47	98.89	95.15	96.03	97.79	98.86
Tree	98.52	99.01	97.33	99.11	94.07	95.98	95.72	**99.90**
Barren	93.61	96.61	87.05	98.06	92.31	95.44	96.13	**99.76**
Pond	**100.00**	**100.00**	98.55	99.59	98.54	98.41	99.32	**100.00**
Other	93.61	97.13	77.33	98.76	89.94	95.52	94.59	**99.13**
OA (%)	96.96	98.28	90.95	98.84	94.73	96.81	97.33	**99.65**
AA (%)	97.00	98.29	91.09	98.84	94.78	96.83	97.36	**99.66**
Kappa	0.9646	0.9799	0.8944	0.9864	0.9385	0.9628	0.9689	**0.9960**

**Table 9:** Comparison of classification results of different classification methods on the Willow County 2021 dataset.

Class	Different Methods							
	SVM	KNN	1DCNN	RNN	ViT	CAF	ViTMLP	LO-MLPRNN
River	95.74	98.31	90.53	97.95	98.61	98.97	99.17	**99.90**
Buildup	97.64	98.72	87.97	97.90	98.16	99.24	98.75	**99.90**
Sugarcane	97.95	**99.02**	92.75	97.85	97.85	97.65	98.76	98.95
Tree	82.14	94.87	97.08	94.43	87.64	91.93	95.35	**98.35**
Barren	77.76	95.51	55.92	91.91	95.17	94.67	90.28	**99.26**
Pond	99.79	99.49	90.18	98.65	98.98	99.42	99.62	**100.00**
Other	84.27	96.94	71.31	93.43	92.96	92.31	94.78	**98.36**
OA (%)	90.71	97.55	82.19	96.00	95.60	96.28	96.66	**99.24**
AA (%)	90.76	97.56	82.26	96.02	95.63	96.32	96.68	**99.25**
Kappa	0.8916	0.9714	0.7922	0.9533	0.9487	0.9566	0.9611	**0.9911**

**Table 10:** Comparison of classification results of different classification methods on the Willow County 2023 dataset.

Class	Different Methods							
	SVM	KNN	1DCNN	RNN	ViT	CAF	ViTMLP	LO-MLPRNN
River	97.92	**99.40**	89.70	98.84	98.84	96.53	99.30	99.27
Buildup	97.05	96.66	89.15	98.59	97.73	99.01	98.98	**99.96**
Sugarcane	98.24	99.22	98.47	98.89	98.40	98.99	99.38	**99.83**
Tree	98.52	99.01	97.33	99.11	98.94	99.40	99.76	**100.00**
Barren	93.61	96.61	87.05	98.06	98.30	98.93	98.36	**99.20**
Pond	**100.00**	**100.00**	98.55	99.59	99.42	**100.00**	99.29	**100.00**
Other	93.61	97.13	77.33	98.76	98.24	97.43	99.01	**99.66**
OA (%)	96.96	98.28	90.95	98.84	98.55	98.60	99.16	**99.71**
AA (%)	97.00	98.29	91.09	98.84	98.56	98.62	99.16	**99.71**
Kappa	0.9646	0.9799	0.8944	0.9864	0.9831	0.9837	0.9902	**0.9966**

**Table 11:** Table of changes in vegetation use types in Willow County from 2019 to 2023.

Class	Area (km^2^)			Area Change Rate (%)		
	2019	2021	2023	2019–2021	2021–2023	2019–2023
River	230.21	211.60	276.64	−8.08	30.74	20.17
Buildup	383.80	391.26	635.19	1.94	62.34	65.50
Sugarcane	443.96	458.48	547.25	3.27	19.36	23.27
Tree	908.91	841.86	1225.16	−7.38	45.53	34.79
Barren	785.82	914.34	438.64	16.35	−52.03	−44.18
Pond	140.27	89.78	78.24	−35.99	−12.85	−44.22
Other	732.37	718.01	424.19	−1.96	−40.92	−42.08

## Data Availability

The data presented in this study are available on request from the corresponding author.

## Abbreviations

The following abbreviations are used in this manuscript:

MLP	Multilayer Perceptron
RNN	Recurrent Neural Network
LSK	Large-scale Selective Convolutional Network
ODC	Omni-Dimensional Dynamic Convolution
GRU	Gated Recurrent Unit
ROI	Region of Interest
SVM	Support Vector Machine
KNN	K-nearest neighbor
ViT	Vision Transformer
OA	Overall Accuracy
AA	Average Accuracy
